# Thematic Analysis of Dyadic Coping in Couples With Young-Onset Dementia

**DOI:** 10.1001/jamanetworkopen.2021.6111

**Published:** 2021-04-15

**Authors:** Sarah M. Bannon, Victoria A. Grunberg, Mira Reichman, Paula J. Popok, Lara Traeger, Bradford C. Dickerson, Ana-Maria Vranceanu

**Affiliations:** 1 Integrated Brain Health Clinical and Research Program, Department of Psychiatry, Massachusetts General Hospital, Harvard Medical School, Boston; 2 Department of Psychiatry, Massachusetts General Hospital, Harvard Medical School, Boston; 3 Frontotemporal Disorders Unit, Department of Neurology, Massachusetts General Hospital, Harvard Medical School, Boston

## Abstract

**Importance:**

A diagnosis of young-onset dementia (YOD) is a life-altering event for both persons with dementia and their spousal caregivers. Dyadic coping (DC) theoretical models acknowledge that dyads cope with stressors as a unit, but these models have yet to be used in YOD.

**Objective:**

To explore the lived experiences of couples managing YOD using an integrated DC model.

**Design, Setting, and Participants:**

This qualitative study recruited couples from a single major medical setting and through social media. Eligibility criteria included cohabitation, 1 partner diagnosed with YOD and able to participate, and both partners willing to participate. Live online video interviews were conducted from March to June 2020.

**Exposures:**

One semistructured interview, which was recorded and subsequently transcribed. Recruitment was stopped once thematic saturation was reached.

**Main Outcomes and Measures:**

Five themes were deductively derived based on the integrated DC framework, including stress communication, positive individual DC, positive conjoint DC, negative individual DC, and negative conjoint DC. Within each theme, subthemes were inductively identified to further characterize couples’ dyadic coping experiences with YOD.

**Results:**

A total of 23 couples were interviewed, comprising persons with dementia (11 women [48%]; mean [SD] age, 61.3 [4.65] years; mean [SD] time from diagnosis, 3.11 [3.85]) years; and spousal caregivers (13 women [57%]; mean [SD] age, 60.5 [5.40] years). Data supported the 5 a priori DC themes and novel subthemes describing couples’ experiences with YOD. Specifically, couples enacted positive conjoint DC by approaching challenges using a teamwork approach to problem solving and relying on collaborative communication. In contrast, couples engaged in negative conjoint DC through mutual avoidance, leading to increased negative communication and conflict. Couples described initially engaging in avoidance and withdrawal to navigate YOD-related stressors. While these strategies provided short-term relief from challenging emotions, they prevented engagement in adaptive coping (eg, acceptance and collaborative problem-solving) to promote long-term adjustment.

**Conclusions and Relevance:**

This study used an evidence-based integrated DC approach to identify the positive and negative coping behaviors of couples managing YOD. To our knowledge, this is the first study to use a DC framework to guide qualitative analysis, and it provides valuable insights into DC strategies used by couples navigating YOD-related stressors. Findings can inform the development of dyadic psychosocial services for couples managing YOD and have implications for other progressive illnesses.

## Key Points

**Question:**

How do couples cope with stressors following a diagnosis of young-onset dementia (YOD) individually and as a dyad?

**Findings:**

In this qualitative study of 23 couples, those managing YOD engaged in patterns of coping consistent with dyadic coping theoretical models. Couples described patterns of stress communication and positive and negative coping behaviors enacted by individual partners and the couple as a unit.

**Meaning:**

The findings of this study could inform the development of dyadic psychosocial services for couples managing YOD and other progressive illnesses.

## Teaser

This qualitative thematic analysis explores the lived experiences of couples managing a young-onset dementia diagnosis using an integrated dyadic coping model.

## Introduction

Young-onset dementias (YODs) are defined by symptom onset prior to 65 years and have atypical symptoms relative to dementias diagnosed at older ages. YOD can be diagnosed in persons in the prime of their lives,^1^ when they are employed, parenting, or supporting older parents^2,3^ and generally, in good health. Persons with YOD (PWDs) are challenged to manage symptoms and their consequences (eg, loss of work and income), navigate medical care,^4,5^ and plan for an uncertain, often rapid symptom progression with no available cure or meaningful treatments.^6^ It is not surprising that both PWDs and their spousal caregivers (SCGs), who provide most informal care, endorse high levels of emotional distress.^7^ However, there are few age-appropriate psychosocial resources for couples managing YOD-related stressors.^8,9^

Couples adjusting to progressive health conditions benefit from strategies that increase mutual understanding and cultivate adaptive coping.^10,11,12^ Individual coping (ie, used by 1 partner to manage a difficult situation) is associated with better health outcomes for both partners.^13^ However, adjustment to illness is associated with both partners’ individual coping efforts and their attempts to manage stressors as a dyad (ie, pair).^14,15,16^

Dyadic coping (DC) models provide a theoretical underpinning for capturing couples’ psychosocial adjustment to serious medical conditions such as YOD. DC models describe how dyads, including couples, cope or respond to stressors in positive and negative ways. They emphasize that stress and coping processes are interdependent and how individual and shared coping patterns affect the dyad. In 2019, Falconier and Kuhn^17^ integrated prominent DC models into a singular conceptually integrated DC framework with the following domains: (1) explicit stress communication (eg, direct, open communication about challenges),^18^ (2) positive or negative DC by 1 partner (eg, how individual positive or negative coping affects the dyad), and (3) positive or negative DC by both partners as a unit^17^ (how shared coping affects the dyad). These frameworks are supported by empirical findings, which demonstrate that stress communication precedes individual coping and DC, which has long-term consequences on the dyad over time.^18^ Prior work with YOD couples has focused on individual positive (eg, SCG adapting communication, PWD accepting diagnosis)^6,19^ and negative (eg, SCG role transitions, PWD avoidance or denial) stress and coping experiences. Given the limited attention to DC, investigation of these processes is warranted.

Applying this integrated DC framework for YOD couples can elucidate modifiable processes to target through dyadic interventions. In fact, dyadic interventions aimed to reduce negative DC and promote positive DC have alleviated emotional distress among couples with many medical conditions (eg, breast cancer, prostate cancer, stroke, acute neurological injury).^20,21,22,23^ To date, very few studies have examined dyadic adjustment to YOD, and no work has explored how this DC framework applies to couples with YOD. Our goal was to understand how couples manage YOD-related stressors individually and together in the context of this DC framework. We sought to understand how DC domains relate to YOD-specific stressors and coping patterns, with the overall goal of using our findings to inform the development of a psychosocial intervention for couples with YOD.

## Methods

### Study Design and Sample

We conducted a qualitative dyadic study to gather detailed insights about couples’ experiences following a YOD diagnosis using a conceptually integrated DC framework.^17^ We recruited couples from the Massachusetts General Hospital Department of Neurology clinics and YOD social media support groups to create a convenience sample. Couples were eligible for inclusion if both partners were older than 18 years, both partners spoke English, 1 partner received a diagnosis of YOD (eg, frontotemporal dementia, Alzheimer disease), and both partners were involved in a cohabitating romantic relationship. Couples were excluded if either partner was unwilling to participate or the neurologist or neuropsychologist on study team determined a PWD’s cognitive impairment too severe to participate in the interview meaningfully. We contacted 38 couples by telephone. Of them, 4 (11%) declined participation, 5 (13%) were ineligible, and 6 (16%) were unresponsive, leaving 23 couples as potential participants. Recruitment stopped when thematic saturation was achieved.^24^ Participants provided written informed consent electronically and participated in interviews between March and June 2020. We followed the Consolidated Criteria for Reporting Qualitative Research (COREQ) reporting guideline, and the Massachusetts General Hospital institutional review board approved study procedures.

### Data Collection

We developed a 60-minute semistructured interview that was informed by the DC framework, prior literature,^25,26,27^ our qualitative systematic review,^6^ and weekly multidisciplinary team meetings (ie, neurologist, neuropsychologist, speech-language pathologist, 3 clinical psychologists, and the spouse of a deceased PWD). A PhD-level female clinical psychologist (S.B.) with expertise in couple and family psychology conducted a one-time joint interview with each dyad over a secure telehealth platform. To begin, the clinical psychologist established rapport and introduced study aims with participants. She received biweekly supervision from the multidisciplinary team. Interview guide included open-ended questions addressing psychosocial stressors and strategies for coping with YOD-related stressors (eAppendix in the Supplement). Audio recordings of interviews were deidentified and transcribed verbatim by trained research assistants.

### Data Analysis

Data analysis was conducted between July and September 2020. We uploaded transcripts to NVivo 12 qualitative data analysis software package (QSR International). We used a hybrid deductive-inductive^28^ approach to thematic analysis. We deductively derived 5 a priori themes from the integrated DC theoretical framework^17^: (1) stress communication, (2) positive individual DC, (3) positive conjoint DC, (4) negative individual DC, and (5) negative conjoint DC. All transcripts were coded independently by 2 members of the research team (M.R. and P.J.P). Then, using a collaborative and iterative inductive approach, we extracted subthemes within each DC theme. Three members of the research team (S.B., M.R., P.J.P.) independently extracted subthemes within DC themes based on prior literature,^6,7,29^ data observations, and collaborative discussions.^28^ Two members (S.B., V.G.) met for in-depth discussions to refine subthemes and resolve inconsistencies until consensus was reached.

## Results

### Demographic Characteristics

Among the 23 couples, 22 (96%) were opposite-sex partners, and 1 couple (4%) was same-sex. The couples had been together for a mean (SD) of 34 (10) years. Couples included 11 women and 12 men PWDs with a mean (SD) age of 61.30 (4.65) years, and 12 women and 10 men SCGs with a mean (SD) age of 60.52 (5.41) years. Couples were predominantly non-Hispanic White individuals (SCG, 21 [91%]; PWD, 20 [87%]) and college-educated. Twelve PWDs (52%) had an Alzheimer disease diagnosis, and symptoms had a mean (SD) onset of 3.11 (3.85) years before study participation. Table 1 displays participant characteristics.

**Table 1.** Participant Characteristics of 23 Couples With a Young-Onset Dementia Diagnosis

Characteristic	Participants, No. (%)	
	SCGs	PWDs
Sex		
Female	13 (57)	11 (48)
Male	10 (43)	12 (52)
Race/ethnicity		
White		
Non-Hispanic or Latino	21 (91)	20 (87)
Hispanic or Latino	0	1 (4)
Asian, non-Hispanic or Latino	1 (4)	0
Multiracial, non-Hispanic or Latino	0	1 (4)
Not reported	1 (4)	1 (4)
Education		
High school or GED	1 (4)	0
Some college or associate’s degree	2 (9)	4 (17)
4-y college or bachelor’s degree	8 (35)	10 (43)
Graduate or professional degree	12 (52)	9 (39)
Diagnosis		
Alzheimer disease		
Atypical	NA	8 (35)
Typical	NA	4 (17)
FTLD		
Behavioral variant	NA	4 (17)
Language variant	NA	2 (9)
Atypical	NA	1 (4)
Prodomal	NA	1 (4)
PSP	NA	2 (9)
Unspecified early-onset dementia	NA	1 (4)
Years in relationship, mean (SD [range])	33.70 (9.65 [13-48])	33.70 (9.65 [13-48])
Age at interview, mean (SD [range])	60.52 (5.41 [49-69])	61.30 (4.65 [54-70])
Age at diagnosis, mean (SD [range])	NA	57.87 (5.33 [45-67])

Abbreviations: FTLD, frontotemporal lobar degeneration; GED, general educational development; NA, not applicable; PSP, progressive supranuclear palsy; PWD, persons with dementia; SCG, spousal caregivers.

### DC Themes and Subthemes

We organized data in 5 central deductive themes determined a priori by the integrative DC framework. We inductively identified 2 to 5 subthemes within each theme. The Figure presents the themes and subthemes and are described in detail in the following sections. Table 2 displays illustrative quotations within DC themes (quotations identified by couple number [1-23] and participant role [PWD, SCG]).

**Figure.**
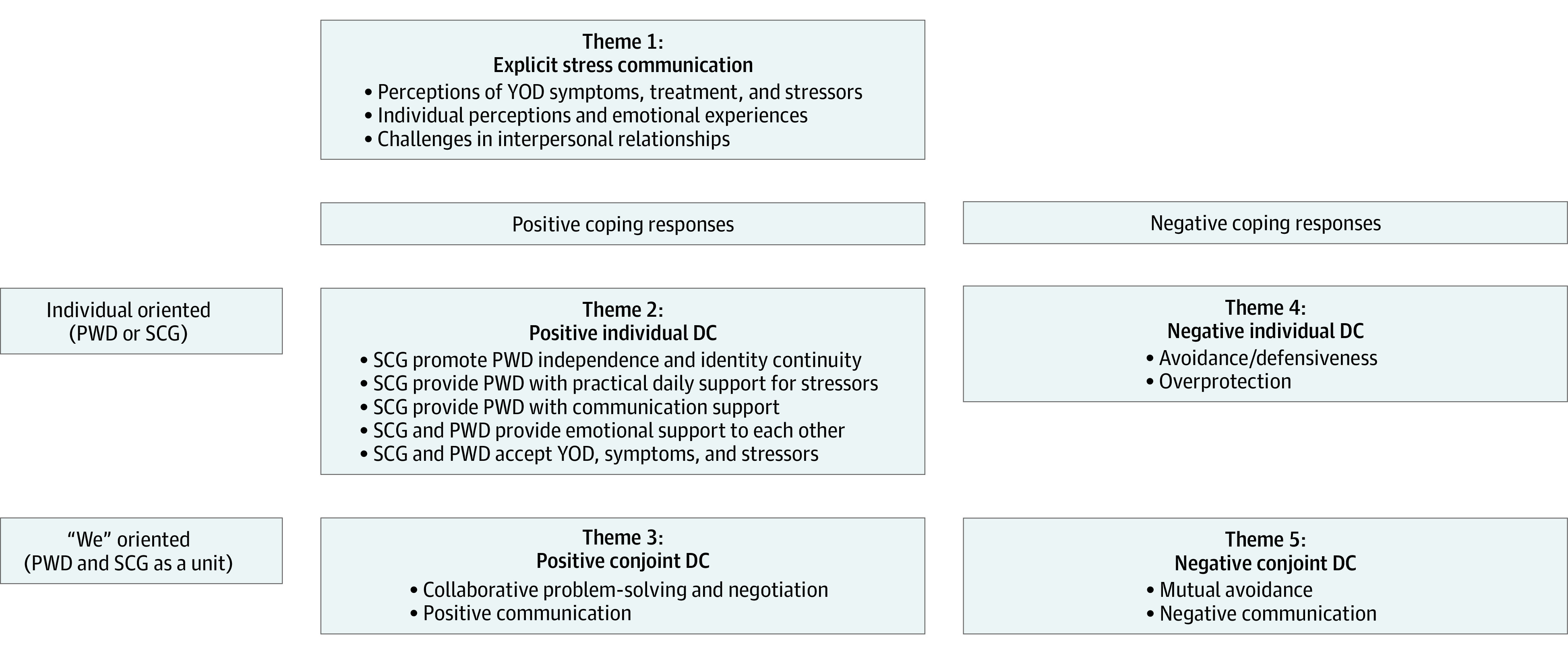
Dyadic Coping (DC) Themes and Subthemes Abbreviations: PWD, patients with dementia; SCG, spousal caregivers; YOD, young-onset dementia.

**Table 2.** Description of Themes and Illustrative Quotations

Theme	Theme definition	Illustrative quotations
Explicit stress communication	Open and specific communication of the experience of stress between partners, including details on an individual’s point of view and emotions with regard to it.	SCG 23: I think oftentimes what happens is it builds up, and it does get to the point, and then when we sit down and we talk through it, it does help.
		SCG 15: I mean I will be honest; I think that since even before his diagnosis with the whole losing things, you know, I definitely get frustrated. And he’ll tell you I get frustrated and, you know, there were times where I would be crying because I felt so bad for snapping at him because I get frustrated. But I think as a couple we kind of come through it. We talk about it. We talk about a lot of things.
Positive individual DC	One partner’s positive response(s) to help the other cope with stressors.	Dyad 13, SCG: I check in with her, I say how do you feel about your driving? And make sure nothing has happened and basically check in with you on that, see how you are feeling. PWD: I find that very enlightening to have him check with me. And not just assume that he knows what it is that is going on. So I think that that was helpful to me, too.
		SCG 15: If [PWD] has a struggle with the way that I am managing things, she obviously comes right out and says it. What I will do is step back and make sure that I try to present it in the best light possible.
		SCG 22: I try to be careful because it’s something where you’re never sure how [PWD] is receiving the information. …To me, it’s helping if I just try to keep mentioning “oh, I’m going to do this now. I’m going to do this now.” That type of thing rather than just going out on an errand or going to another end of the house and maybe he won’t remember the conversation or know where I am. Or sometimes when I’m going out on an errand, I’ll write a note on the kitchen table just in case he forgets and thinks “where’d she go?” You know, that kind of thing.
Positive conjoint DC	Ways in which partners positively cope with a shared or dyadic stressor together.	PWD 21: Yes, and our new idea about if somebody’s having stress or whatever, we stop. We say let’s talk about this a little bit later instead of getting sad. That kind of coping thing as well.
		SCG 15: Once we had agreement. Once we had an understanding, I guess that things were much different, or starting to become much different. It became, in my opinion, it became more of a team effort to try to get it figured out. And then there’s been ongoing communication about every symptom. Everything that has come up since.
		SCG 21: Sometimes it takes us a while for the person to say I just really need to go take a walk and we can finish this conversation later or sometimes the other person has to say maybe you should go take a walk and we can finish this conversation later. We found that’s been helpful.
Negative individual DC	One partner's negative response(s) to the other partner's stress.	SCG 2: For me it’s tough because I wanna talk about how scared I am, but I feel like I’m burdening him even more (cries).
		PWD 23: One thing important to me is I don’t want to put more of a burden on her with any of this … my only concern like I certainly want to help, but I don’t want it by helping put more on her.
Negative conjoint DC	Ways in which partners negatively cope with a shared or dyadic stressor together.	Dyad 21, SCG: Maybe just logistical type things in terms of—is it best for you if you’re going to drive off to a strange place or is it better for me? PWD: Well, that’s never been an issue, so I don’t know why you’re making it one now. I’m not really sure. SCG: I’m not making it an issue. PWD: But you’re concerned? SCG: I mean we haven’t talked about it I guess. PWD: Yeah. So, I had no idea that you thought that I was a danger to myself in terms of driving or anything. And I’m only saying that because I want to let you know that I would tell you that. I would articulate that to you, you know what I mean? So that’s why I’m surprised you’re saying that because I would tell you that. SCG: Maybe it’s just because I worry about everything.
		SCG 11: Yes, but she would get really upset, so I became cautious about how I talked to her about it. That is kind of what got us into marriage counseling is that she was angry a lot, and I’d bring up things and she would just get really angry.
		SCG 12: We just make this decision and keep going and in fact sometimes we don’t even talk about it, because it raises anxiety, so…

Abbreviations: DC, dyadic coping; PWD, persons with dementia; SCG, spousal caregivers.

### Theme 1: Explicit Stress Communication

This theme pertains to communication about the content and experience of stress between partners. We identified 3 subthemes, including perceptions of YOD symptoms and stressors, individual perceptions and emotional experiences, and interpersonal relationship challenges. Couples communicated about individual and shared YOD-related stressors, including managing symptom progression, navigating medical care, and daily tasks. After diagnosis, couples shared concerns about stigma, how to disclose YOD diagnosis to their social network (eg, children, friends), and managing loved ones’ emotional reactions. Couples communicated about how SCGs can best assist PWDs in safely participating in daily tasks (eg, cooking) given progressive symptoms. As time progressed, couples learned to openly communicate their individual perceptions of YOD, emotional experiences, and changing relationships with others. Both partners described stressors related to their relationship, typically stemming from different understanding and preferences about symptoms, role changes, and future plans (Table 2).

### Theme 2: Positive Individual DC

We extracted 5 subthemes that describe how 1 partner positively responded to the other partner’s stressors. Three of these subthemes involve SCGs promoting PWDs’ independence and identity, providing PWDs with practical support, and providing PWDs with communication support. Two of the subthemes involved both SCGs and PWDs individually providing emotional support to each other and accepting YOD symptoms and stressors.

There were many ways in which SCGs provided support to PWDs. SCGs promoted identity continuity for PWDs (eg, saying statements such as he’s still him). SCGs also adapted to PWDs’ progressive communication difficulties by helping PWDs participate in social interactions and communication. For example, SCGs would ask clarifying questions and assist PWDs with word-finding during conversations with family and friends. SCGs modified their communication by using shorter sentences, repeating statements, and giving PWDs adequate time to finish sentences. SCGs provided PWDs with practical support for daily activities and in medical settings. For example, SCGs provided PWDs with detailed instructions for chores (eg, buying groceries) and were active in managing PWDs’ medical care (eg, scheduling appointments, communicating with clinicians, identifying resources).

Both PWDs and SCGs learned to accept disruptions in communication. They described picking their battles during disagreements about symptoms and care. Both PWDs and SCGs helped provide support to their partner regarding overwhelming emotions or concerns about the future (eg, using statements like it’s alright, it’s a bad day, and tomorrow will be a good day). PWDs and SCGs noted that proactively checking in with their partners about challenging situations helped promote effective communication and maintain intimacy.

### Theme 3: Positive Conjoint DC

Two subthemes described ways in which partners positively coped with a shared or dyadic stressor together, which included collaborative problem-solving and negotiation and positive communication. Couples explained that approaching problems collaboratively (eg, planning for the future or establishing short-term routines) was positive for their relationship. Many couples took a problem-solving approach to slow symptom progression by engaging in positive health behaviors (eg, exercise, nutrition, mindfulness, relaxation). They found that enacting a team effort was beneficial for managing symptoms and facilitating PWDs’ participation in daily tasks. Couples who were comfortable sharing their difficulties endorsed more positive DC behaviors. Couples with more interactive conversations involving open communication, thoughts or feelings, and impressions of ongoing discussions suggested that communication helped promote relationship quality and prevent conflict. Couples learned to use several specific communication strategies, including collaboratively deciding when to have challenging conversations, being willing to pause discussions if one or both partners become emotionally overwhelmed, and building difficult discussions into ongoing routines (eg, dinners, walks).

### Theme 4: Negative Individual DC

Two subthemes were identified that addressed the ways in which 1 partner negatively responded to another’s stressors: avoidance or defensiveness and overprotection. Most couples described the initial adjustment to YOD as filled with negative (maladaptive) coping attempts (eg, avoidance of sharing difficult emotions and individual concerns). SCGs described engaging in overprotective or controlling behaviors, such as closely monitoring or offering excessive support as PWDs complete tasks. They also restricted PWDs from engaging independently in activities (eg, cooking, using the internet/cell phone, exercising). Overprotective or controlling behaviors contributed to reduced self-esteem and feelings of sadness and frustration for PWDs. In turn, these experiences increased relationship strain and conflict for both partners. In addition, partners did not want to burden each other, so they avoided discussions about challenging topics (eg, YOD prognoses, long-term care planning), which led to increased strain given the lack of attention to practical concerns. Partners’ desires to protect each other led to misguided attempts to support one another (eg, attempting to mind-read, making assumptions about type and level of support needed by each partner). At times, PWDs viewed support from SCGs as unhelpful or overprotective, which contributed to relationship conflict. Conversely, SCGs found it difficult to navigate current symptoms, plan for progressive symptoms, and discuss future care needs when PWDs were defensive or avoidant of acknowledging symptoms.

### Theme 5: Negative Conjoint DC

Two subthemes emerged that described ways in which partners negatively coped with a shared or dyadic stressor together that included mutual avoidance and negative communication. Couples shared that negative interactions stemmed from mutual avoidance or heightened reactivity to YOD-related challenges. Couples felt unprepared for conversations about the future given the uncertainty of symptom progression. They also felt unsure about how to discuss changing roles, responsibilities, and the relationship impact of YOD. After diagnosis, many couples described walking on eggshells around each other. They felt uncomfortable or unwilling to discuss their individual stress, emotions, and perspectives on how to navigate YOD symptoms and care. Some couples explained that mutual avoidance led to an accumulation of frustration, which culminated in negative communication (eg, partners lashing out at each other). During the joint interviews, many couples had difficulty providing examples of these negative coping behaviors, which some couples attributed to mutual avoidance of communicating about stressful experiences.

## Discussion

This qualitative study aimed to understand how PWDs and their SCGs navigate challenges associated with YOD as a unit. We used an integrated DC theoretical model^17^ to guide the thematic analysis of joint interviews among 23 couples with YOD. We described how dyads approached and coped with YOD-related stressors, including coping strategies that can promote or inhibit positive adjustment for PWDs and SCGs. To our knowledge, this is the first study that used an integrated DC framework to conduct qualitative thematic analysis and investigate dyadic adjustment to YOD. Findings will inform novel dyadic interventions for couples coping with YOD and the application of the DC framework to other illnesses.

Couples described negative individual and conjoint DC strategies (eg, avoidance and overprotection), particularly in the time shortly after diagnosis. Avoidance initially provided short-term relief by serving as an escape from difficult emotions, symptoms, role changes, and future planning. However, these avoidant behaviors ultimately created more challenges for couples. Prior YOD work indicates that avoidant coping can lead to reductions in positive coping behaviors, lower use of psychosocial services and social support, and delays in planning for the future.^26,30,31,32,33^ With other serious illnesses, avoidance can be a barrier to a teamwork approach to illness management and, in turn, can contribute to declines in mental and physical outcomes and relationship satisfaction.^17^ Our findings also revealed that SCGs tended to become overprotective as the cognitive abilities of PWDs declined. SCGs’ overprotective behaviors were meant to protect PWDs from harm; however, these behaviors often led to defensiveness from PWDs and further relationship strain. The lack of explicit communication seemed to lead to misunderstandings between partners, thereby isolating each of them from each other. Our findings are consistent with prior literature that has found associations of imbalanced partner support with lower quality of life and higher depression symptoms for support recipients.^34,35^ Outside of YOD research, caregivers’ overprotection has been associated with more distress, worse physical health, reduced medical adherence for care-receiving partners, and lower relationship satisfaction and intimacy for both partners.^36^ Although avoidance and overprotection were both positively intentioned, they created more relationship conflict for couples.

Some couples described learning to communicate more explicitly about stress over time, which led to more adaptive coping. Chronic illness literature highlights how clear, open, and frequent communication about daily stressors^18,37,38,39,40,41^ can enhance couples’ quality of life and partner support.^42,43^ Viewing an individual condition as a we-illness has been associated with greater relationship quality and positive adjustment (eg, among couples managing HIV, cancer).^35,44,45,46,47^ We found that couples adjusted to increased dependency through a teamwork approach^6^ involving positive communication, collaborative problem solving, and negotiation. Interestingly, couples endorsed greater variety in positive DC strategies than negative DC strategies, suggesting that there are several different potential strategies to target to promote adaptive coping. Although positive DC strategies generally increased over time, some couples continued to engage in negative coping.

Together, findings underscore the imminent need for tailored interventions that target DC for couples with YOD. Dyadic interventions developed for other neurologic conditions (eg, stroke, acute neurologic injury) have used education and skills practice to successfully reduce emotional distress and preserve well-being for both dyad members.^22,23^ These interventions could be adapted to help promote positive DC strategies for couples shortly after YOD diagnosis. Given the rapid advances in biomarker research that enable clinicians to make earlier and more confident YOD diagnoses, PWDs are being identified when they exhibit less cognitive impairment.^1^ Therefore, there is increasing potential to involve PWDs in interventions with their SCGs when they are still able to meaningfully engage. Programs that seek to overcome barriers to positive DC could enhance explicit dyadic communication about individual and shared stressors. For example, teaching emotion regulation skills may help couples cope with conflicting and difficult emotions and better communicate experiences and needs. Sharing their individual distress with each other may help normalize experiences and promote collaborative problem-solving. In addition, interpersonal effectiveness skills could help couples learn a shared language to help them adjust to a new normal (eg, use we rather than I, give PWDs time to respond). Skills training could also be used to help them plan their time together, focus on valued behaviors, and find time to recharge as a couple. Such approaches can help reduce emotional distress, enhance teamwork, and maintain the PWDs’ independence and dignity, thus empowering PWDs to take an active role in care and promote the well-being of SCGs. Findings are relevant for couples coping with YOD and a variety of progressive illnesses, particularly those affecting memory and cognition.

### Strengths and Limitations

Our study has several strengths. We used a theory-driven approach to guide thematic analysis of dyadic interviews, which allowed us to identify adaptive coping strategies that may inform intervention development. Furthermore, our approach to data analysis drew from prior literature about DC to identify superordinate themes (eg, deductive techniques) and allowed for flexibility in identifying subthemes related to DC in YOD (eg, inductive techniques). Finally, we focused on both positive and negative DC. This approach is consistent with recent guidelines in dementia clinical research to identify modifiable factors linked to resiliency after diagnosis.

Our study was limited because we conducted joint interviews of PWDs and SCGs to characterize DC processes in YOD. Therefore, individual perspectives were not fully captured, given that partners may have shared more openly or directly if interviewed alone. Our findings may not be generalizable to other PWD-caregiver dyads (eg, parental, sibling) or to couples coping with later-onset dementias given the differences in the DC of couples based on their life stage.^48^ Our sample consisted of couples who predominantly resided in a northeastern metropolitan region in the United States, were White individuals, and were opposite-sex partners. In addition, our sample was largely homogenous in terms of relationship length, mean age, and level of education. Thus, the perspectives gathered may not reflect those of more diverse samples of couples, including other geographic regions or those with varying individual and relationship characteristics. Finally, our goal was to provide an overview of DC experiences in couples after YOD diagnosis. Therefore, our study did not characterize relationships among themes, nor did we explore patterns of coping over time. Future studies are needed to investigate associations among themes described in this study and DC patterns that couples experience throughout the YOD disease continuum.

## Conclusions

To our knowledge, this is the first qualitative investigation of DC after YOD diagnosis. Our methods may apply to couples’ experiences after the onset of chronic medical conditions to identify how couples cope with such stressors individually and as a unit. We hope that these findings will inform the development of dyadic interventions for PWDs and their caregivers to positive adjustment to YOD-related stressors.

## Notes

Supplement. **eAppendix**. Interview Script
