# Supplementary material for: Thematic Analysis of Dyadic Coping in Couples With Young-Onset Dementia
Source: JAMA Netw Open. 2021 Apr 15;4(4):e216111. doi: 10.1001/jamanetworkopen.2021.6111 (PMC8050740; doi:10.1001/jamanetworkopen.2021.6111)
Supplement: Supplement. — eAppendix. Interview Script [file jamanetwopen-e216111-s001.pdf]

Supplemental Online Content

Bannon S, Grunberg VA, Reichman M, et al. Thematic analysis of dyadic coping in couples with young-onset dementia. *JAMA Netw Open*. 2021;4(4):e216111. doi:10.1001/jamanetworkopen.2021.6111

eAppendix. Interview Script

This supplemental material has been provided by the authors to give readers additional information about their work.

© 2021 Bannon SM et al. *JAMA Network Open.*

eAppendix. Interview Script

My name is ____________________ and I am_________________________. I am part of the research team that is interested in learning about the experiences and challenges of adjusting to and living with a diagnosis of _____________. We are interested in developing a program to help with understanding people's journeys before and after the diagnosis, how the symptoms and diagnosis impact the relationship between a couple, and what people need in order to adjust to and cope with the diagnosis and the impact on their lives. We have some ideas based on our clinical experience and previous research, but we want to learn about your experiences as a patient and care-partner so that we can ensure that the program we will develop is going to be helpful and relevant.

We also recognize that you each have your own viewpoint about these topics. This may be the first time you are discussing these viewpoints, and you may or may not agree with each other on the topics of discussion. We hope you feel comfortable being honest even if you disagree about the answers to certain questions. Additionally, we are interested in both of your individual perspectives, including where your experiences or opinions are different. So, throughout the interview, we want you to share from your own experiences, and we will make sure to give both of you the opportunity to share on each topic.

Intro/warm-up.

To start, we would love to learn a little bit about both of you and your family. Could each of you please tell me your name, what you do for a living, and what are some activities you enjoy?

[*Note: Ensure that both patient and care-partner provide responses.***]**

EARLY SYMPTOMS & DIAGNOSIS (

Early symptoms

We like to start by learning more about what you each first noticed that prompted the evaluations you went through to get to the diagnosis. Can you each tell me about the earliest symptoms you noticed?

Diagnosis

Tell me about your experience of getting a diagnosis.

What was the impact of receiving the diagnosis?

Needs after receiving diagnosis

What were your greatest needs immediately after receiving the diagnosis?

With respect to these needs, what resources were provided to you after the diagnosis?

CHALLENGES FACED SINCE DIAGNOSIS

Symptoms and changes in functioning

What are the most noticeable or troubling symptoms that you have experienced since the time of diagnosis?

How have your changes in functioning impacted you?

Emotionally, how do you feel about your symptoms and the changes in functioning you are experiencing?

Stigma

Are you open with your friends and family about the ______ diagnosis?

Have you experienced any stigma related to your diagnosis?

Understanding diagnosis and progression

What is your understanding of the___ diagnosis?

What is your understanding about the how this condition will affect you both in the future?

How are you getting information about this diagnosis?

***Is there anything about the diagnosis that you don't understand well, and wish you understood better?**

***Is there anything about the diagnosis, that you have shied away from wanting to learn about or think about?**

Recognizing that these conditions have a lot of variability, do you have any questions that you would like answered more concretely if your doctors were better able to make personalized predictions?

***How does your understanding of your diagnosis impact you emotionally?**

Preparedness for the future

What are the biggest practical concerns or future changes that you are worried about?

How have your thoughts about the future changed since receiving the diagnosis?

***What emotions come up when you think or plan about the future?**

CHANGES IN RELATIONSHIP

Social relationships

We will ask questions about your relationship with each other in a moment, but at first let's talk about your relationships with other people.

How do you feel about your support network and relationships in general?

© 2021 Bannon SM et al. *JAMA Network Open.*

(*If dyad has children)* How has it been for your children?

<u>Relationship with partner prior to diagnosis</u>

Tell me a little about your relationship together. What was your relationship like before the diagnosis?

What was your communication like with each other before the diagnosis?

<u>Changes in relationship with partner after diagnosis</u>

Now I would like to ask you about how your relationship has changed since receiving the diagnosis, including any challenges you have experienced. This might be a difficult topic to discuss together. However, I really appreciate your honesty and any insight you are able to share with me about how your relationship has changed, and what has been hard or uncomfortable. Since we are looking to develop a program for couples, it is extremely important for us to know what couples find hard or uncomfortable.

What changes have you observed in your relationship, if any?

In general, how do you feel the communication between the two of you has been since the diagnosis?

What are some challenges you face with respect to communicating?

<u>COPING</u>

<u>Coping with symptoms and changes in functionality</u>

What are some strategies that have helped you deal with the symptoms you are experiencing?

*** Communicating with each other and others?**

<u>Coping with emotions</u>

How much do you feel that the difficult emotions (*reference what was disclosed earlier)* you experience impact you on a day-to-day basis?

How do you cope with these difficult emotions?

<u>Coping with expected changes in relationship</u>

What are some changes in your relationship with each other that you have experienced since the diagnosis?

(*To care-partner)* Have you begun to need to do things to take care of your partner as a result of this condition?

<u>Meaningful/pleasurable activities</u>

© 2021 Bannon SM et al. *JAMA Network Open.*

What activities do you enjoy doing the most these days? Together or individually?

Coping by planning for the future

Making plans for the future, including both short-term and long-term, is a way of coping.

How have you been making plans for the future?

What resources have you used to help you plan?

Do you think you could use more guidance about planning for the future when living with this condition, or do you feel confident about how you are making plans?

***How do you communicate/plan to communicate about your needs and wants?**

> **Probe: In terms of care?**
>
> **Probe: About changes in your relationship with your partner/ as a couple?**

Do you write down your ideas related to planning? Do you use any other systems, formal or informal, for keeping track of your plans or thoughts/feelings about things you need to plan for?

***If there are some difficult emotions associated with making these plans, how do you support each other through these?**

WRAP-UP

Many thanks for participating.

© 2021 Bannon SM et al. *JAMA Network Open.*
